# Immigrant Status and Social Ties: An Intersectional Analysis of Older Adults in the United States

**DOI:** 10.1007/s10903-024-01644-z

**Published:** 2024-11-21

**Authors:** Sameera S. Nayak, Christine A. Mair, Suliyat O. Adewuyi

**Affiliations:** 1 Department of Sociology, Anthropology and Public Health, University of Maryland Baltimore County, 1000 Hilltop Circle, Baltimore, MD 21250 USA; 2 Center for Health, Equity, and Aging, University of Maryland Baltimore County, Baltimore, MD 21250 USA

**Keywords:** Immigrant, Social ties, Older adults, Social support, Nativity

## Abstract

Diverse social ties are critical facilitators of well-being among older adults. Social ties might be especially important for aging immigrants who face multiple social and economic vulnerabilities over the life course. We investigated social ties (e.g., partners, children, other family, and friends) by immigrant status among older adults in the United States (U.S.). Data come from the 2018 Health and Retirement Study (*N* = 4,006), a national sample of older adults in the U.S. We used multivariable logistic regression to compare social ties (e.g., partners, children, other family, and friends) by immigrant status. We further explored interactions with sex and race/ethnicity. Older immigrants are more likely to report that they can rely a lot on their partners (aOR = 1.84, 95% CI 1.27, 2.68) but less likely to rely on friends (aOR = 0.72, 95% CI, 0.55, 0.94) compared to non-immigrants. Older immigrants are also less likely to meet frequently with friends (aOR = 0.66, 95% CI, 0.51, 0.86) and with other family (aOR = 0.71, 95%, CI, 0.55, 0.91) compared to non-immigrants. Lastly, older immigrant men are significantly less likely to meet with friends compared to non-immigrant men (aOR = 0.48, 95% CI, 0.32, 0.73). As the older population in the U.S. continues to diversify and immigrant older adults navigate their support options, older immigrants–especially men–may be at risk for less variation in their social support options, particularly from extended family members and friends.

## Introduction

The United States (U.S.) is home to a large aging immigrant population; those 65 and older make up 17% of the 43 million foreign-born individuals, and an estimated 1.2 million undocumented adults are 55 or older [1, 2]. In 2021, the median age of the foreign-born population was ten years older than the median age of the U.S.-born population, 47 years vs. 37 years, respectively [1]. These numbers are rapidly increasing, and the U.S. Census Bureau projects that the U.S. will have 22 million older immigrants by 2060 [3]. Alongside the general health and well-being risks arising as a function of aging, older immigrants face multiple social and economic vulnerabilities related to their migration patterns and legal status. Older immigrants are often less financially secure in retirement due to reduced Social Security benefits resulting from a shorter time in the U.S. workforce, lower wages than U.S.-born counterparts, and delayed retirement [4, 5]. Many immigrants who migrated in late life might have limited English proficiency and face difficulties with social integration and acclimatizing to a new country [6]. Later-life migration has risen since U.S. immigration policy was restructured in 1965 to prioritize family reunification. In 2022, over 132,000 immigrants received lawful permanent residency (“green cards”) through their adult children [7].

Social ties and social support are critical facilitators of well-being among older adults [8–10]. The literature on social ties and immigration is mixed; some studies find evidence that immigrants tend to have stronger social ties and higher perceived social support than non-immigrants, and some have found that migration limits social ties and leads to reduced social support [11–15]. Contextualizing social ties among older immigrants is more complex because they might have amassed transnational family, friends, and other social networks across the life course. The extant research on social ties among older immigrants in the U.S. has either been qualitative [16–18] or quantitatively focused on specific ethnic communities, the living arrangements of older immigrants, and the role of social support in predicting a variety of outcomes [19–26]. Existing studies on older immigrants have also focused heavily on nuclear family ties and have yet to explore the extent to which extended family members and friends may be necessary for a healthy aging experience. Because of the ever-changing nature of federal and state immigration policy in the last decade that has had direct impacts on migration patterns, there is a need for research on the social networks of older immigrants with more recent data to better capture the experiences of immigrants in the U.S. today. At the time of writing, we were unable to find any existing studies that comprehensively compared the familial and friendship-based social ties of older immigrants and non-immigrants using recent national data.

Additionally, social ties vary markedly by sex and race/ethnicity, underscoring the need to examine the relationship between immigrant status and aging using an intersectional lens [27–29]. Women, for example, maintain more extensive and diverse social networks compared to men [30]. Black women, in particular, are more likely to rely on non-partnership relationships compared to White women [31]. Older adults from socially disadvantaged groups have also been shown to have higher network turnover [32]. Rather than treating older immigrants as a homogeneous group, it is necessary to understand how nativity, sex, and race/ethnicity might intersect to have heterogeneous effects on older immigrants’ social ties and networks. This intersectional approach to immigrant health has been recommended in the modern literature [33].

This study examines the relationship between immigrant status and social ties with partners, children, other family members, and friends. Specifically, we explore the extent to which being an immigrant predicts the frequency of interaction with social ties and the extent of reliance on these social ties in a national sample of older adults in the U.S. We also examine whether sex and race/ethnicity moderate these associations.

## Methods

### Data Source

Data come from the 2018 (Wave 14) of the Health and Retirement Study (HRS). The HRS is a nationally representative longitudinal panel study of adults aged 50 and older in the United States. It is produced and distributed by the University of Michigan with funding from the National Institute on Aging (grant number NIA U01AG009740) [34]. Our sample draws from the RAND HRS data source (Version 1, 2020) as well as the core HRS data from the Psycho-Social Module in the Leave Behind Questionnaire. The RAND HRS is a cleaned version that is produced by the RAND Center for the Study of Aging, with funding from the National Institute on Aging and the Social Security Administration [35]. The HRS is administered in English and Spanish. Because the motivation of this paper is to examine reliance on and contact with social ties, we analyzed data from 2018 prior to the onset of “physical distancing” during the COVID-19 pandemic. Additional details for HRS methodology are provided elsewhere [36] and on the HRS website: https://hrs.isr.umich.edu/.

### Measures

#### Exposure

We ascertained immigrant status based on respondents’ nativity (U.S.-born or foreign-born).

#### Outcomes

The main outcome variables were the probability that a respondent: (1) can rely on a social tie, and (2) meets frequently with a social tie. Respondents were asked about several social ties (partner, child or children, other family members, and friends) to determine how much they could “rely on them if you have a serious problem,” which we dichotomized to compare “a lot” compared to less than that (combining “some”, “a little”, or “not at all”). Next, respondents were asked how often they “meet up (include both arranged and chance meetings)” (in-person) with each of these social ties (coresident partnerships were not included in this question). We dichotomized this measure to compare meeting up once or twice a month (combining “three or more times a week”, “once or twice a week”, and “once or twice a month”) compared to less frequently (combining “every few months,” “once or twice a year”, and “less than once a year or never”).

#### Interactions

Our main interaction variables included sex (1 = female, 0 = male) and race/ethnicity (White Non-Hispanic, Black Non-Hispanic, Other Non-Hispanic, and Hispanic).

#### Covariates

Control variables included education (1 = has some college; 0 = high school degree or less), categorical age (50–59 years, 60–69 years, 70–79 years, and 80 + years), self-rated health (1 = excellent or good), depression (1 = depression), household income (deciles), household wealth (deciles), and whether or not a respondent reported that they have a partner, a child or children, other family, and friends.

### Statistical Analysis

We first examined descriptive statistics of all variables by immigrant status and conducted t-tests of differences between groups. Next, we conducted multivariable logistic regression analyses predicting (1) the probability that respondents can rely on partners, child(ren), other family, and friends and (2) how frequently respondents meet with child(ren), other family, and friends. We analyzed each of these social ties separately. Only respondents with a coresident partner were included in the models examining reliance on a partner (*N* = 2632). Similarly, only respondents who have children (*N* = 3604), have other family (*N* = 3732), and have friends (*N* = 3534) were included in each respective model. Multivariable models controlled for education, age, self-rated health, depression, household income, household wealth, and whether or not they had a partner, a child or children, other family, and friends. Additionally, we examined interaction effects between immigrant status and sex, as well as immigrant status and race/ethnicity, to determine if sex and race/ethnicity moderate the associations between immigrant status and social ties. Because the HRS sampling includes spousal dyads, all regression models are clustered by household to adjust standard error estimates. Analyses were performed in SAS 9.4.

## Results

### Sample Characteristics

Sample characteristics, stratified by immigrant status, reveal patterns of similarity and dissimilarity (Table 1). Compared to non-immigrants, immigrants in our sample were more likely to be partnered (74% compared to 65%), slightly more likely to have children (92% compared to 90%), less likely to report having other family (90% compared to 94%), and less likely to report having friends (83% compared to 89%). Among those who are partnered, about 81% of non-immigrants and 83% of immigrants reported that they can rely a lot on their partners. Among those who have child(ren), about 65% of non-immigrants and 63% of immigrants report that can rely a lot on their child(ren). Immigrants were more likely to report they can rely a lot on other family members (45% compared to 41% among non-immigrants) and were markedly less likely to report they can rely on friends (31% compared to 42% among non-immigrants). Immigrants and non-immigrants were somewhat similar in terms of meeting with children (61% vs. 65%) and other family ties (44% vs. 43%). However, frequency of contact with friends revealed differences, with nearly 74% of non-immigrants versus 61% of immigrants reporting that they socialize with friends frequently (1–2 times a month). The immigrant subsample was less likely to have some college (48% versus 58%), skewed younger, was less likely to report being in excellent or good self-rated health (32% versus 42%), more likely to report depression (17% versus 9%), and fell into the lower deciles of household income (4.03 versus 4.90) and wealth (3.87 versus 4.77) compared to the non-immigrant subsample.

**Table 1:** Descriptive statistics of all variables and T-Tests of differences (Health and Retirement Study, Wave 14, 2018)

	Non-Immigrant		Immigrant		Difference
	*(N = 3502; 87.42%)*		*(N = 504; 15.28%)*		*t-test*
	N	%	N	%	p-value
Female	3502	59.79%	504	58.53%	0.5892
White Non-Hisp	3502	73.90%	504	21.83%	< 0.0001
Black Non-Hisp	3502	17.90%	504	7.14%	< 0.0001
Other Non-Hisp	3502	5.05%	504	33.93%	< 0.0001
Hispanic	3502	5.28%	504	59.33%	< 0.0001
Some College	3502	57.71%	504	48.02%	< 0.0001
Household Income (deciles)	3502	4.80	504	4.03	< 0.0001
Household Wealth (deciles)	3502	4.77	504	3.87	< 0.0001
Aged 50–59	3502	23.24%	504	34.92%	< 0.0001
Aged 60–69	3502	33.30%	504	39.29%	0.008
Aged 70–79	3502	25.87%	504	16.67%	< 0.0001
Aged 80+	3502	17.59%	504	9.13%	< 0.0001
Good SRH	3502	41.66%	504	32.34%	< 0.0001
Depressed	3502	9.19%	504	17.46%	< 0.0001
Has Partner	3502	64.51%	504	74.01%	< 0.0001
Has Partner + Can Rely	2259	81.32%	373	82.57%	0.5634
Has Child(ren)	3502	89.66%	504	92.06%	0.0674
Has Child + Can Rely	3140	64.52%	464	62.50%	0.3963
Has a Child + Frequent Visits	3140	64.90%	464	61.42%	0.1436
Has Other Family	3502	93.58%	504	90.28%	0.0175
Has Other Family + Can Rely	3277	41.47%	455	45.49%	0.1032
Has Other Family + Frequent Visits	3277	43.33%	455	43.96%	0.8015
Has Friend(s)	3502	89.01%	504	82.74%	0.0004
Has a Friend(s) + Can Rely	3117	41.80%	417	30.94%	< 0.0001
Has a Friend + Frequently Visits	3117	73.92%	417	61.15%	< 0.0001

*Note*: Sample size is smaller when restricted to only respondents who have stated social tie

### Associations Between Immigrant Status and Social Ties

Table 2 includes the results of multivariable logistic regression models predicting the probability that older adult respondents can rely on their social ties, controlling for covariates. Among coresident partnered older adults, immigrant older adults were more likely to report being able to rely on their partner “a lot” compared to non-immigrants (aOR = 1.84, 95% CI 1.27, 2.68). Conversely, immigrant older adults were less likely to report being able to rely “a lot” on friends (aOR = 0.72, 95% CI 0.55, 0.94) compared to non-immigrants. Table 3 depicts the probability of meeting up frequently with social ties (at least 1–2 times a month), controlling for covariates. Immigrant older adults were less likely to meet up in person with other family (aOR = 0.71, 95% CI 0.55, 0.91) compared to non-immigrant adults. Similarly, immigrant older adults were less likely to meet up in person with friends (aOR = 0.66, 95% CI 0.51, 0.86) compared to non-immigrants. We did not observe any differences in the probability of meeting up with children among immigrant and non-immigrant older adults. Complete results from all multivariable models are presented in Tables 2 and 3.

**Table 2:** Adjusted logistic regression results predicting probability can rely on social ties

Social Tie	Probability Respondent Can Rely (A Lot) on Partner, Child, Other Family, or Friends															
	*Partner (N = 2632)*				*Child (N = 3604)*				*Other Family (N = 3732)*				*Friends (N = 3534)*			
	OR	Lower	Upper	Sig.	OR	Lower	Upper	Sig.	OR	Lower	Upper	Sig.	OR	Lower	Upper	Sig.
Immigrant	1.84	1.27	2.68	**	1.15	0.88	1.50		1.11	0.86	1.42		0.72	0.55	0.94	*
Female	0.51	0.41	0.63	***	1.30	1.13	1.50	***	1.41	1.23	1.62	***	1.56	1.35	1.79	***
Black Non-Hisp	0.63	0.47	0.85	**	0.88	0.71	1.09		1.17	0.97	1.42		0.83	0.67	1.02	
Other Non-Hisp	0.66	0.45	0.97	*	0.74	0.56	0.98	*	1.17	0.90	1.54		1.02	0.76	1.36	
Hispanic	0.84	0.57	1.22		1.13	0.85	1.51		1.33	1.02	1.74	*	0.79	0.59	1.05	
Has Some College	0.92	0.73	1.15		0.93	0.80	1.10		0.78	0.67	0.91	**	1.00	0.86	1.17	
Household Income	1.07	1.02	1.12	**	0.98	0.95	1.02		1.04	1.01	1.08	*	1.01	0.98	1.05	
Household Wealth	1.06	1.01	1.12	*	1.00	0.97	1.04		0.99	0.96	1.02		1.00	0.97	1.03	
Age in 60s	1.08	0.83	1.40		1.18	0.97	1.42		0.93	0.77	1.11		0.93	0.77	1.12	
Age in 70s	1.40	1.02	1.93	*	1.55	1.24	1.93	***	1.05	0.85	1.28		0.86	0.70	1.06	
Age in 80s	1.31	0.87	1.97		3.92	2.96	5.18	***	1.22	0.96	1.54		0.86	0.67	1.09	
Good SRH	1.39	1.11	1.74	**	1.35	1.15	1.58	***	1.26	1.09	1.46	**	1.49	1.29	1.73	***
Depressed	0.68	0.48	0.95	*	0.70	0.55	0.89	**	0.89	0.70	1.12		1.01	0.78	1.30	
Has Partner					0.96	0.80	1.16		0.81	0.69	0.96	*	0.77	0.65	0.92	**
Has Child(ren)	1.15	0.71	1.86						0.71	0.57	0.90	**	0.72	0.57	0.91	**
Has Other Family	1.64	1.12	2.40	*	1.42	1.06	1.90	*					1.13	0.85	1.51	
Has Friend(s)	1.60	1.19	2.15	**	1.47	1.18	1.82	***	1.69	1.34	2.14	***				

*Note*: OR = Odds Ratios, lower/upper = lower bound and upper bound of 95% confidence intervals; *=*p* < 0.05, **=*p* < 0.01, ***=*p* < 0.001

**Table 3:** Adjusted logistic regression results predicting probability meets frequently with social ties

Social Tie	Probability Respondent Meets Frequently (At Least 1-2x/Month) with Child, Other Family, or Friends											
	*Child (N = 3604)*				*Other Family (N = 3732)*				*Friends (N = 3534)*			
	OR	Lower	Upper	Sig.	OR	Lower	Upper	Sig.	OR	Lower	Upper	Sig.
Immigrant	0.96	0.75	1.24		0.71	0.55	0.91	**	0.66	0.51	0.86	**
Female	1.15	1.00	1.33		1.10	0.96	1.27		0.96	0.82	1.12	
Black Non-Hisp	0.65	0.53	0.80	***	1.22	1.01	1.48	*	0.61	0.49	0.76	***
Other Non-Hisp	0.64	0.49	0.83	***	1.06	0.81	1.38		0.92	0.69	1.23	
Hispanic	0.97	0.74	1.27		1.96	1.51	2.56	***	0.84	0.63	1.12	
Has Some College	0.98	0.84	1.14		0.74	0.64	0.86	***	1.07	0.91	1.27	
Household Income	0.98	0.94	1.01		1.00	0.96	1.03		0.99	0.95	1.03	
Household Wealth	1.01	0.98	1.05		1.01	0.98	1.04		1.05	1.02	1.09	**
Age in 60s	0.85	0.70	1.03		0.82	0.69	0.97	*	1.20	0.98	1.46	
Age in 70s	0.86	0.70	1.06		0.95	0.77	1.15		1.25	0.99	1.57	
Age in 80s	0.94	0.74	1.20		0.79	0.63	1.00		1.02	0.78	1.32	
Good SRH	0.94	0.81	1.09		0.92	0.79	1.06		1.24	1.05	1.46	**
Depressed	0.90	0.71	1.13		0.96	0.77	1.21		0.78	0.60	1.01	
Has Partner	1.06	0.89	1.27		0.94	0.79	1.11		0.73	0.60	0.88	***
Has Child(ren)					1.11	0.88	1.39		0.88	0.67	1.15	
Has Other Family	1.35	1.03	1.78	*					1.00	0.73	1.36	
Has Friend(s)	1.24	1.00	1.53		1.64	1.31	2.05	***				

*Note*: OR = Odds Ratios, lower/upper = lower bound and upper bound of 95% confidence intervals; *=*p* < 0.05, **=*p* < 0.01, ***=*p* < 0.001

### Interaction Effects

**Fig. 1:**
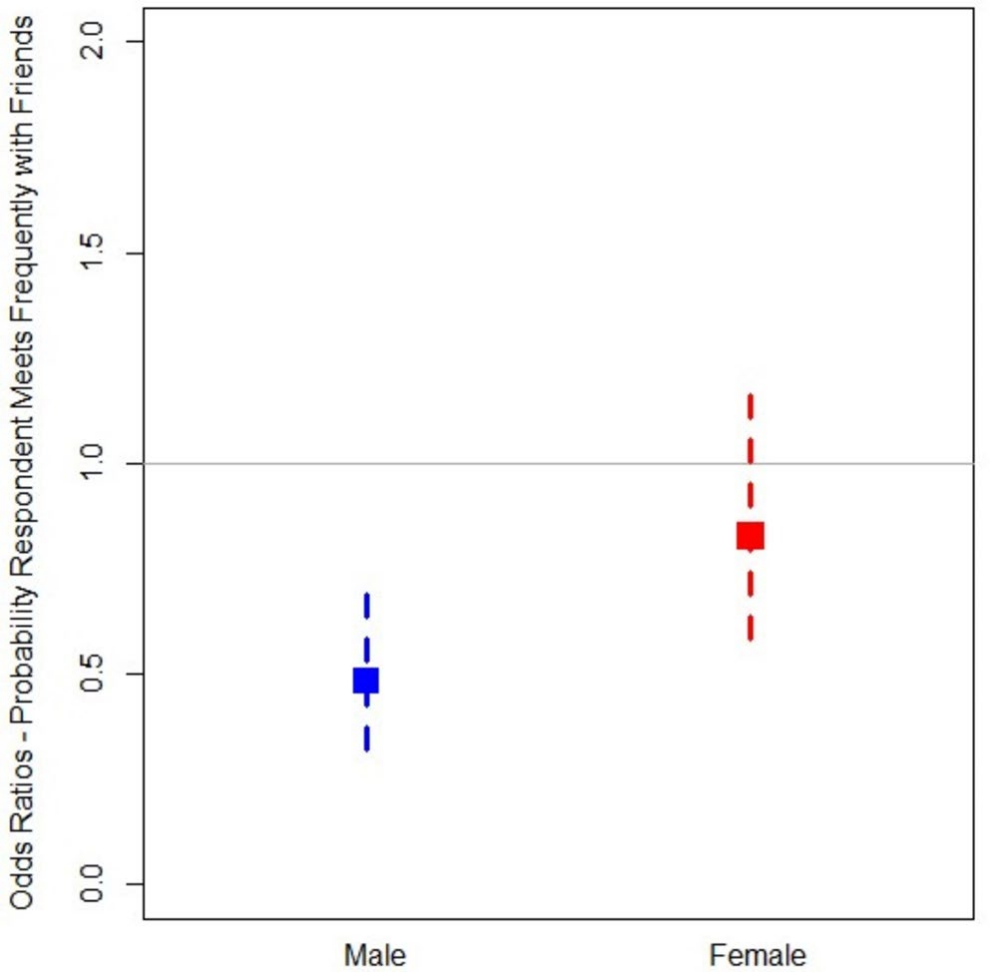
Association between immigrant status and frequency of contact with friends by sex

We tested 14 interaction models to examine potential effect modification by sex (immigration status*sex) and race/ethnicity (immigration status*race/ethnicity) on the associations between immigration status and social ties. These models identified only one statistically significant interaction term (immigrant*female), predicting the probability of interacting frequently with friends. Specifically, we found that the statistically significant association between being an immigrant older adult and having a lower likelihood of interacting frequently with friends is present only for male immigrants. To illustrate this interaction, we ran this model separately by sex. In these stratified models (Fig. 1), we found no association between immigrant status and the likelihood of frequently interacting with friends among female immigrants. Male immigrant older adults, however, were substantially less likely to interact with friends (aOR = 0.48, 95% CI 0.32, 0.73) compared to non-immigrant male older adults.

## Discussion

In this national sample of older adults living in the U.S., we found differences in the patterns of social ties between immigrants and non-immigrants. Results suggest that immigrant older adults have greater reliance on their coresident partners compared to their non-immigrant counterparts. In other words, aging immigrants depend more heavily on their partnerships for support compared to aging non-immigrants. There could be several reasons for this observed difference. Depending on the migration mechanism, immigrant older adults might have migrated with their partners and thus have a stronger shared sense of closeness as they navigated a new life in the U.S. Older immigrants who are partnered with non-immigrants may also rely more on their partners because their partners might serve as the primary gateway to their larger social network. These findings could have both positive and negative effects for immigrant older adults. On one hand, a strong sense of reliance might be a proxy for a strong partnership, which can have positive health and well-being effects in aging populations [37–39]. On the other hand, if older immigrants are overly reliant on their partners and their social networks, they might experience more severe grief, financial strain, and loneliness in the event of the death of their partner [40, 41]. This might be exacerbated for women who have longer life expectancies and are more likely to experience widowhood.

Results from this study also highlighted that immigrant older adults were less likely to have frequent in-person meetings with other family members and friends. One explanation for this could be that immigrant older adults are more likely to have transnational familial ties and friendships, making frequent meetups financially and logistically challenging [18, 42]. Furthermore, travel can become more difficult for older adults with comorbidities or age-related functional limitations. Although the growth of technological innovation has increased the ability to have frequent remote contact (e.g., phone calls, video calls, emails, social media), some research suggests that in-person contact is especially beneficial for the well-being of older adults [43]. A lack of in-person contact has been associated with reductions in happiness and increases in depressive symptoms and loneliness in older adults [43, 44]. Our findings suggest that older immigrant adults might thus be more vulnerable to these mental health challenges because of their reduced in-person interaction with family members and friends.

Finally, we found that sex moderated the association between immigrant status and meeting with friends, with older immigrant men being less likely to meet with their friends compared to women and non-immigrant men. Previous literature indicates that older men have smaller social networks than older women [45, 46], and this pattern may be exacerbated among older immigrant men for a variety of reasons. Immigrant men may have more limited social ties outside of the family due to the need for additional paid work, high levels of familism and reliance on family ties, geographic isolation due to relocation, or difficulty becoming socially integrated into a new country [47]. Future research should explore this group more thoroughly to consider the unique risks faced by older immigrant men.

### Limitations

Despite the strengths of the analysis, this study includes several limitations to consider for future work. First, this analysis is cross-sectional and therefore cannot determine causality or account for changes in social ties over time. Future researchers should examine how immigrants’ social ties change over the life course from their first arrival in the U.S. This could provide a better understanding of the mechanisms that might be driving the findings from this study. Next, our models did not include a consideration of legal status (e.g., citizenship) or time spent in the U.S., which may influence the formation of social ties. This is because these variables typically do not apply to non-immigrants and could not be included in our analytic models. Moreover, the subsample of immigrant respondents who completed the social ties questionnaire was too small to stratify and precluded us from conducting subgroup analyses only on the immigrant subsample. We were also unable to stratify by race/ethnicity for these same reasons. It is possible that additional subgroup differences exist by race/ethnicity, but our sample size did not have sufficient power to detect these differences. The analyses were further limited by the dataset itself. HRS is only available in English and Spanish. This means that individuals who speak other languages are excluded from participation. Given that the immigrant population in the U.S. is increasingly varied, HRS likely may not capture the full diversity of immigrant experiences. These findings might not apply to immigrant groups who are entirely non-English and non-Spanish speaking. On one hand, it is possible that these groups experience more severe social isolation and exclusion due to their limited language ability in English or Spanish. On the other hand, it could be that these groups have built tighter community networks to compensate for their broader social exclusion. Although the HRS does not have the data to test these hypotheses, future research with different datasets could explore these issues. Next, the HRS collects nativity data, whether participants are born in the U.S. or not, and data on the U.S. state of birth. However, we were unable to locate any options for more granular data on immigrant respondents’ country of birth. The only option we located was a subjective country-of-origin identification variable that was asked only of Hispanic participants. Consequently, we were unable to explore more nuanced cultural and social differences based on immigrants’ countries of origin. Cultural norms around family networks and friendships vary worldwide, but these variations are not captured in our analysis. Country of origin could also be an important variable to understand the formation of social ties because of the disparate immigration histories of different groups. For example, immigrants from countries who primarily arrived as refugees or asylum seekers are likely to have different social ties than those who arrived for education or labor-based reasons. Future research with more linguistically diverse samples and with more granular information on country of origin is warranted to better contextualize social ties among older immigrants.

## Conclusions

Despite these limitations, the results of this study suggest clear patterns by immigrant status in terms of older adults’ social ties, and indicate a possible enhanced risk of isolation (lack of friend meetings) among older immigrant men. This study is the first, to our knowledge, to compare social ties among immigrant and non-immigrant older adults in a recent, nationally representative sample of older adults from an intersectionality perspective and therefore, provides a substantial contribution to the literature on the social needs of the rapidly diversifying population of older adults in the United States.
